# Fissurectomy versus lateral internal sphincterotomy in the treatment of chronic anal fissures: no advantages in terms of post-operative incontinence

**DOI:** 10.1007/s10151-023-02780-8

**Published:** 2023-03-16

**Authors:** Roberta Tutino, Casimiro Nigro, Flavia Paternostro, Rosa Federico, Giacomo Lo Secco, Gaetano Gallo, Mauro Santarelli, Gianfranco Cocorullo, Sebastiano Bonventre

**Affiliations:** 1 General Surgery 3 O.U., Molinette Hospital, University Hospital Città Della Salute E Della Scienza di Torino, Turin, Italy; 2 Department of Surgery, Università Degli Studi Di Roma “Tor Vergata”, Rome, Italy; 3 Medical Oncology O.U., Università Campus Biomedico Di Roma, Rome, Italy; 4 University Hospital “P. Giaccone” of Palermo, Palermo, Italy; 5 Department of Surgical Sciences, University of Torino, Turin, Italy; 6 Department of Surgical Sciences, Sapienza University of Rome, Rome, Italy; 7 Department of Surgical, Oncological and Stomatological Disciplines, University of Palermo, Palermo, Italy

**Keywords:** Anal fissure, Lateral internal sphincterotomy, Fissurectomy, Anal canal/surgery, Faecal incontinence

## Abstract

**Purpose:**

The standard treatment for chronic anal fissures that have failed non-operative management is lateral internal sphincterotomy. Surgery can cause de novo incontinence. Fissurectomy has been proposed as a sphincter/saving procedure, especially in the presence of a deep posterior pouch with or without a crypt infection. This study investigated whether fissurectomy offers a benefit in terms of de novo post-operative incontinence.

**Methods:**

Patients surgically managed with fissurectomy or lateral internal sphincterotomy for chronic anal fissures from 2013 to 2019 have been included. Healing rate, changes in continence and patient satisfaction were investigated at long-term follow-up.

**Results:**

One hundred twenty patients (55 females, 65 males) were analysed: 29 patients underwent fissurectomy and 91 lateral internal sphincterotomy. Mean follow-up was 55 months [confidence interval (CI) 5–116 months]. Both techniques showed some rate of de novo post-operative incontinence (> +3 Vaizey score points): 8.9% lateral internal sphincterotomy, 17.8% fissurectomy (*p* = 0.338). The mean Vaizey score in these patients was 10.37 [standard deviation (sd) 6.3] after lateral internal sphincterotomy (LIS) and 5.4 (sd 2.3) after fissurectomy Healing rate was 97.8% in the lateral internal sphincterotomy group and 75.8% in the fissurectomy group (*p* = 0.001). In the lateral internal sphincterotomy group, patients with de novo post-op incontinence showed a statistically significant lower satisfaction rate (9.2 ± 1.57 versus 6.13 ± 3; *p* = 0.023) while no differences were present in the fissurectomy group (8.87 ± 1.69 versus 7.4 ± 1.14; *p* = 0.077).

**Conclusions:**

Lateral internal sphincterotomy is confirmed as the preferred technique in term of healing rate. Fissurectomy did not offer a lower rate of de novo post-operative incontinence, but resulted in lower Vaizey scores in patients in whom this occurred. Satisfaction was lower in patients suffering a de novo post-operative incontinence after lateral internal sphincterotomy.

## Introduction

Anal fissure is the second most frequent cause for a proctologic consultation, after haemorrhoidal disease. The disease is more prevalent in young people, with both sexes equally affected, and is associated with an impairment of social and working life.

The aetiology is still unclear, various anatomical considerations have been postulated, such as reduced vascular supply in the posterior midline region of the anal canal and the fixity of the anoderm to the muscle in this zone, resulting in both more frequent lacerations and difficulty of healing in this area.

An infective aetiology has also been proposed: persistent bacterial overgrowth caused by the colonization of the anal crypts, leading to difficult healing of the fissure [1]. This theory focuses the attention on the treatment of the crypts.

It is classified as acute if appeared recently (less than 6 weeks) and chronic if it has been present for a longer time and has developed a distinctive appearance, such as a proximal papilla, a perianal skin tag with or without a deep crypt and fibrotic borders [2]. Acute and chronic fissures are commonly associated with anal sphincter spasms. Traditionally, the treatment of chronic anal fissure (CAF) has been lateral internal sphincterotomy (LIS). In the last two decades, new options for reducing the tone of the internal anal sphincter have been proposed. Today, topical calcium channel blockers, glycerine trinitrate and botulinum toxin represent the first-line treatments, but in chronically non-healing fissures, surgery remains the main option.

There are also subgroups of patients in which the anal pressure is normal or low, and who may not benefit from sphincter tone reduction. These fissures can be associated with obstructive defaecation syndrome and dysfunctional toilet habits, and/or with regular anal intercourse; where clinically unclear, anorectal physiology/manometry could/should be considered pre-operatively.

LIS has success rates exceeding 90%, but a major risk for post-operative faecal incontinence of up to 9.2% [3, 4]. Generally, anal sphincter damage is effectively compensated but incontinence can also develop several years after surgery [5], as in women who have had sphincter disruption during childbirth who are more likely to experience delayed anal incontinence [6].

Modern proctology has increasingly focused on the preservation of anatomy, to avoid the post-operative sequelae of surgical incision of the ano-rectal structures. This occurs for anal fistulas and hemorrhoids, among others [7]. To offer a sphincter-sparing technique, and according to the infective theory, fissurectomy has been proposed. The treatment comprises the coagulation of the fissure and excision of the crypt, the hypertrophic papilla and the borders to create a healthy environment for a restitutio ad integrum, also removing the site of possible bacterial overgrowth.

In our department, fissurectomy as alternative to LIS has been used since 2015.

No definitive data are present in literature about the benefits of this technique and its advantages over traditional LIS in CAF.

The aim of this study was to compare the results of fissurectomy and LIS in terms of cure rate, continence preservation and patient’ satisfaction.

## Materials and methods

This retrospective study was conducted at the department of Emergency Surgery of the University hospital ‘P. Giaccone’ of Palermo. No ethical approval was required. Informed consent was obtained from all individual participants included in the study.

Patients surgically managed for CAF from 2013 to 2019, identified by the diagnostic code on admission of International Classification of Diseases (ICD)-9: 565.0, have been included.

Data were collected from medical records and electronic archives. Follow-up was performed with outpatient visits and telephone interviews.

Demographic data including age, number and type of previous perineal surgery, type and duration of symptoms were extracted from medical records and electronic archives. Pre-operative bowel function including frequency of defecation, urgency and anal continence was taken from patient records and confirmed in the follow-up interviews.

Patients were divided into two groups according to the surgical technique used: fissurectomy or LIS.

Homogeneity in terms of mean age, sex and previous perineal surgery between the groups was verified.

In our practice, fissurectomy comprises the removal of fissure borders, hypertrophic papilla, sentinel pile, the coagulation of the base of the fissure and, if present, the lay open of a submucosal fistula (crypt) without sphincter section. The LIS technique is a closed one, performed with scissors up to a half of the internal sphincter. All patients underwent local anaesthesia and mild sedation as previously described [8]. All the interventions have been performed under the direct supervision of an expert proctologist (G.C.).

Both techniques have been used according to surgeon preference, with fissurectomy usually but not exclusively the preferred treatment in patients with normal/low anal resting tone. At follow-up, healing rate, continence and patient’ satisfaction were analysed.

Healing rate was defined as the resolution of symptoms without the need for further surgical interventions.

Anal continence was evaluated using the Vaizey score, which ranges from 0 (normal continence) to 24 (severe incontinence) [9], assuming as minimally important change modifications of 3–5 [10] from the previous value. Occurrence of de novo incontinence was registered and compared in both groups.

The relationships among de novo post-operative incontinence and gender, previous ano-rectal surgery, age over 60 years and healing were analysed.

Patient satisfaction was determined using a simple numerical scale from 0 (not satisfied) to 10 (completely satisfied).

### Statistical analysis

Descriptive data are presented as parametric and non-parametric. Comparisons between pre-operative and post-operative functional scores was performed using the paired *t*-test or Wilcoxon’s rank sum test. The Mann–Whitney *U* test was used to evaluate patient satisfaction. A *p*-value of < 0.05 was considered statistically significant. Statistical analysis was conducted using SPSS software (SPSS, Chicago, Illinois, USA) and MedCalc Statistical Software (MedCalc Software, Ostend, Belgium).

## Results

One hundred twenty patients [55 females, 65 males; average female age 47.35 (sd 15) years; average male age 53.43 (sd 12.7) years] who underwent surgery between 2013 and 2019 were analysed. Twenty-eight more patients were unavailable at follow-up and were excluded.

Twenty-eight patients [average age 52.3 (sd 14.4) years] underwent fissurectomy and 89 patients LIS [average age 50.27 (sd 13.98) years].

There were no statistically significant differences in mean age, number of females or prior perineal surgery between patients who underwent LIS or fissurectomy (Table 1).

**Table 1:** Patients’ characteristics in the treated groups

	LIS (89)	Fissurectomy (28)	*p*-Value
Age, years [mean (sd)]	50.9 (13.98)	52.62 (14.54)	0.403
Females [*n* (%)]	42 (46.67%)	13 (44.83%)	0.863
Previous perineal surgery [*n* (%)]	15 (16.67%)	7 (24.14%)	0.367

Fifteen patients (16.5%) in the LIS group and five patients (17.3%) in the fissurectomy group had some degree of pre-operative incontinence.

Mean follow-up was 55 months (CI 5–116 months).

Healing rate was 97.8% in the LIS group and 75.8% in the fissurectomy group (*p* = 0.001). Only one patient who had previously undergone LIS and was not cured subsequently underwent fissurectomy, without showing changes in continence.

The mean pre- and post-operative Vaizey scores are presented in Table 2.

**Table 2:** Average Vaizey score, pre-operatively and at follow-up

Mean Vazey score	Pre-operative	1 month follow-up	1 year follow-up	*p*-Value
Diathermy	0,551,724,138	1,285,714	0.84	0.845
				0.638
LIS	1,168,539,326	1,382,022	1,059,524	0.191
				0.752

The mean Vaizey score was not statistically different at 1 month or 1 year post-operatively for either LIS or fissurectomy [(LIS: after 1 month p = 0.845, after 1 year p = 0.638); (fissurectomy: after 1 month p = 0.191; after 1 year p = 0.752)].

In both treatments, patients with pre-operative incontinence did not show a worsening of function. On the contrary, 7.14% of the patients in the fissurectomy group and 8.9% in the LIS group improved after surgery.

The rate of patients in whom continence improved, remained unchanged or worsened is reported in Table 3.

**Table 3:** Patients with pre-operative incontinence and post-operative continence status at follow-up

	Pre-operative incontinence [% (*n*)]		Post-operative changes in Vaizey score [% (*n*)]			
			Improved (> −3)	Unchanged	Worsened (> +3)	De novo (> +3)
Fissurectomy (28)		16.5% (15)	7.14% (2)	75% (21)	17.8% (5)	17.8% (5)
LIS (89)		17.3% (5)	8,9% (8)	84.2% (75)	8.9% (8)	8.9% (8)

De novo post-operative incontinence (> +3 Vaizey points) occurred in 8.9% of patients who underwent LIS and 17.8% of those who underwent fissurectomy (*p* = 0.338). The mean Vaizey score in these patients was 10.37 (sd 6.3) after LIS and 5.4 (sd 2.3) after fissurectomy.

There was no relationship between age over 60 years and post-operative incontinence in the LIS group (*p* = 0.148) nor in the fissurectomy group (*p* = 0.576).

There was no relationship between gender and post-operative incontinence in the LIS group (*p* = 0.328) nor in the fissurectomy group (*p* = 1.001).

There was no relationship between previous ano-rectal operation and post-operative incontinence in the fissurectomy group (*p* = 0.154), nor in the LIS group (*p* = 0.631).

There was no relation between healing and post-operative incontinence in the LIS group (*p* = 0.827) nor in the fissurectomy group (*p* = 1.000).

Mean post-operative patient satisfaction was 9.2 (sd 1.57) in the LIS group and 8.87 (sd 1.69) in the fissurectomy group. The satisfaction rate decreased in both groups in patients with de novo post-operative incontinence (LIS 6.13, sd 3, *p* = 0.023; fissurectomy 7.40, sd 1.14, *p* = 0.077).

## Discussion

Fissurectomy is being considered as an alternative to LIS ,with the idea that this sphincter-saving technique may offer a reduction in the incidence of post-operative incontinence.

Patients with CAF show some degree of pre-operative incontinence, possibly related to the disease. In the proposed series, this was 16.7% among all treated patients.

Interestingly, in both treatments, the patients with pre-operative incontinence did not show a worsening of their function; on the contrary, 7.14% in the fissurectomy group and 8.9% in the LIS group showed a benefit from the treatment.

Contrary to the initial hypothesis, we found a de novo incontinence rate of 17.8% in the fissurectomy group and 8.9% in the LIS group.

The differences in the global changes in anal continence were not statistically significant in either group. In clinical practice, 9% more patients with de novo incontinence should be considered in the fissurectomy group. Although, as described, patients in the fissurectomy group showed lower Vaizey scores.

As in the present series, Ammari et al. suggest that a mild degree of incontinence could be a feature of the CAF itself rather than a consequence of LIS (27.5% in their series). All patients with pre-operative incontinence reported improvement in mild incontinence. Excluding patients with overt incontinence, they found mild post-operative incontinence in 10% of patients [11].

Aigner reported a historical series of 162 patients in whom, despite a higher recurrence rate in fissurectomy compared with LIS (8% versus 2%), fissurectomy showed a benefit in terms of any incontinence (2% versus 5%) [12].

As expected, also in the present study, the cure rate was higher in patients who underwent LIS (97.8% versus 75.8%).

Zeitun et al. presented a study on 50 patients who underwent fissurectomy with a cure rate of 93.6%, healing time of up to 10.3 ± 4.96 weeks and no statistically significant changes in Vaizey score [13].

Schornagel et al. also reported a recurrence rate of 11.6% after fissurectomy but no de novo post-operative incontinence in patients with an intact sphincter who were pre-operatively continent. The only patient with a de novo post-operative Vaizey score of 14 points had previously undergone LIS [14].

Levin et al. demonstrated that internal sphincterotomy may contribute or precipitate anal incontinence in the long term [15].

In contrast, Mousavi et al., comparing LIS with fissurectomy in 62 patients, reported 6.2% faecal incontinence after fissurectomy and none after LIS, and 96.9% versus 100% healing rate for fissurectomy and LIS, respectively [16].

Similar data are reported by Saeed et al., who found transient post-operative incontinence for flatus in 6.97% and faecal incontinence in 4.65% of patients after fissurectomy, compared with none in the LIS group [17].

Due to these conflicting data, despite the fact that surgery may be appropriate without a trial of pharmacological treatment after failure of conservative therapy, as indicated by the ‘Practice parameters for the management of anal fissure’ [4], incontinence is a life-long risk and serious concern to both patients and surgeons, and even with the use of sphincter-saving techniques, a stepwise approach would be appropriate.

Analysing the factors related to the onset of de novo incontinence, no correlation was found with gender, age over 60 years, previous anorectal surgery or healing in either group.

Therefore, it is very difficult to adequately select patients, and a full explanation of possible changes in continence must be provided, including that continence already seems affected in CAF and sometimes improves after surgical treatment.

The results obtained in this study partially support the idea, because LIS is still considered the standard method in the treatment of CAF, but they leave a place for fissurectomy in clinical practice as it seems to offer a lower degree of post-operative incontinence and better satisfaction rates.

The present study offers data on an ongoing question in modern proctology.

This was a retrospective analysis, and the data need to be confirmed by prospective randomized studies.

The healing rate was statistically higher in the LIS group. Post-operative incontinence was higher in the fissurectomy group but with lower Vaizey scores than in the LIS group; however, no statistical differences were found.

Therefore, we cannot state that there is a clear advantage of fissurectomy over LIS in the treatment of CAF.

## Data Availability

The datasets generated during the current study are available from the corresponding author on reasonable request.
